# Aortic Valve Calcium Score Is Associated With Acute Stroke in Transcatheter Aortic Valve Replacement Patients

**DOI:** 10.1016/j.jscai.2022.100349

**Published:** 2022-05-12

**Authors:** Michael Foley, Kerry Hall, James P. Howard, Yousif Ahmad, Manisha Gandhi, Samir Mahboobani, Joseph Okafor, Haseeb Rahman, Nearchos Hadjiloizou, Neil Ruparelia, Ghada Mikhail, Iqbal Malik, Gajen Kanaganayagam, Nilesh Sutaria, Bushra Rana, Ben Ariff, Edward Barden, Jonathan Anderson, Jonathan Afoke, Ricardo Petraco, Rasha Al-Lamee, Sayan Sen

**Affiliations:** a National Heart and Lung Institute, Imperial College London, London, United Kingdom; b Surgery, Cardiovascular and Cancer Division, Imperial College Healthcare NHS Trust, London, United Kingdom; c Yale School of Medicine, Yale University, New Haven, Connecticut

**Keywords:** Aortic stenosis, aortic valve calcium score, computed tomography, stroke, transcatheter aortic valve replacement

## Abstract

**Background:**

Transcatheter aortic valve replacement (TAVR) is the treatment of choice for patients with severe aortic stenosis who are at a moderate or higher surgical risk. Stroke is a recognised and serious complication of TAVR, and it is important to identify patients at higher stroke risk. This study aims to discover if aortic valve calcium score calculated from pre-TAVR computed tomography is associated with acute stroke in TAVR patients.

**Methods:**

We conducted a retrospective, observational cohort study of 433 consecutive patients undergoing TAVR between January 2017 and December 2019 at the Hammersmith Hospital.

**Results:**

This cohort had a median age of 83 years (interquartile range, 78-87), and 52.7% were male. Fifty-two patients (12.0%) had a history of previous stroke or transient ischemic attack. Median aortic valve calcium score was 2145 (interquartile range, 1427-3247) Agatston units. Twenty-two patients had a stroke up to the time of discharge (5.1%). In a logistic regression model, aortic valve calcium score was significantly associated with acute stroke (odds ratio [OR], 1.26; 95% confidence interval [CI], 1.01-1.53; *P* = .02). Acute stroke was also significantly associated with peripheral arterial disease (OR, 4.32; 95% CI, 1.65-10.65; *P* = .0018) and a longer procedure time (OR, 1.01; 95% CI, 1.00-1.02; *P* = .0006).

**Conclusions:**

Aortic valve calcium score from pre-TAVR computed tomography is an independent risk factor for acute stroke in the TAVR population. This is an additional clinical value of the pre-TAVR aortic valve calcium score and should be considered when discussing periprocedural stroke risk.

## Highlights

• Stroke is a significant complication of transcatheter aortic valve replacement.
• Computed tomography can quantify aortic valve calcification prior to intervention.
• Aortic valve calcium on CT was associated with post-TAVR stroke.
• This could inform discussions of individual patient stroke risk prior to TAVR.

## Central Illustration

**Figure:**
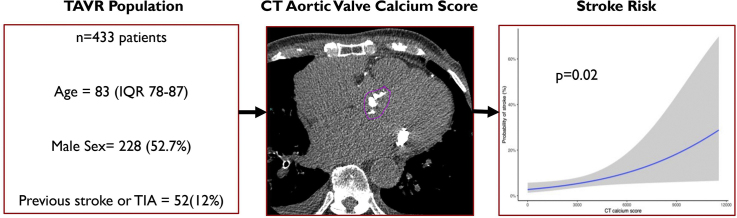

## Introduction

Transcatheter aortic valve replacement (TAVR) has emerged as the treatment of choice for patients in need of aortic valve replacement, who have moderate or higher surgical risk.^1^ Recent trials have demonstrated that TAVR is noninferior to surgical aortic valve replacement (SAVR), even in candidates with a low surgical risk.^2^ In view of this, the worldwide utilisation of TAVR is likely to further increase.

In large trials and TAVR registries, stroke has emerged as an uncommon but serious risk of TAVR, with similar rates reported for TAVR and SAVR.^3^, ^4^, ^5^ Predictors of increased stroke risk after TAVR include baseline clinical factors such as the presence of peripheral arterial disease (PAD) and a history of transient ischemic attack, as well as procedural events such as balloon valvuloplasty post dilatation and valve dislodgement or embolization.^6^

Modern TAVR programmes use preprocedural computed tomography (CT) scanning to establish vascular access routes, annular anatomy, and valve size.^7^ CT has demonstrated utility in predicting post-TAVR outcomes, with left ventricular outflow tract (LVOT) calcification on CT being associated with increased risk of stroke and right coronary cusp calcification being associated with increased pacemaker risk.^8^ CT prior to TAVR also quantifies aortic valve calcium, with a score generated from sequential 3-mm axial noncontrast slices. This score can lend support to a diagnosis of severe aortic stenosis when echo parameters are equivocal.^1^^,^^9^

Aortic valve calcium score has already been demonstrated to correlate well with the severity of aortic valve stenosis.^9^ Echocardiographic and CT studies have shown that a more calcified aortic valve is a predictor of more rapid progression to clinically significant aortic valve stenosis.^10^^,^^11^ In a small TAVR cohort of 64 patients, aortic valve calcium score was an independent predictor of mortality at 1 ​year.^12^ Large prospective observational studies have assessed the prognostic value of aortic valve calcium in non-TAVR populations. It has not been associated with an increased risk of stroke, after correction for other risk factors, although it has been associated with an increased risk of cardiac death and resuscitated cardiac arrest.^13^, ^14^, ^15^

Whether the aortic valve calcium score calculated from the preprocedural TAVR CT scan can predict a higher risk of periprocedural stroke is unknown.

## Methods

### Data collection

We conducted a retrospective observational cohort study with consecutive patients undergoing a TAVR procedure at the Hammersmith Hospital between January 2017 and December 2019. Patients who could not have an accurate aortic valve calcium score calculated because of previous SAVR or TAVR were excluded. Patients who had a TAVR valve implanted in the mitral position were also excluded.

Patient baseline demographic, clinical, and procedural data were collected from the hospital clinical records. Acute stroke was defined as a neurological deficit of vascular origin, lasting more than 24 ​hours with concomitant radiological evidence of new intracerebral hemorrhage or infarct, occurring between the start of their TAVR procedure up until the point of hospital discharge. We additionally included patients with symptoms lasting <24 ​hours where there was imaging evidence of new hemorrhage or infarction or the stroke resulted in death in <24 ​hours according to the Valve Academic Research Consortium 2 definition.^16^ Stroke data were collected from the clinical record. PAD was defined according to the Society of Thoracic Surgeons/American College of Cardiology definition.^17^

**Figure 1:**
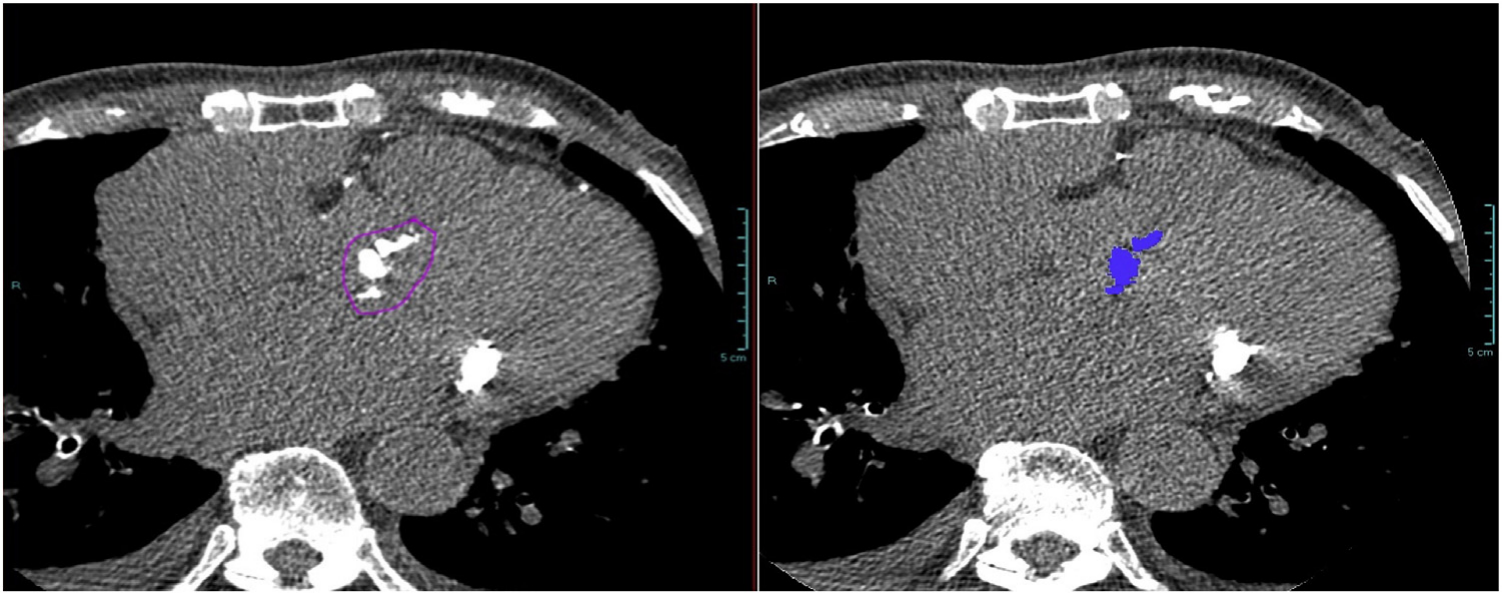
An example 3-mm axial slice from a calcium score sequence of the pre-TAVR CT from a patient in the cohort. CT, computed tomography; TAVR, transcatheter aortic valve replacement.

Aortic valve calcium scores were collected prospectively from the preprocedural TAVR CT. CT scans were undertaken at a voltage of 120 ​kV and included noncontrast sequences. Aortic valve calcium score calculation was performed using an inbuilt software program on the Vue PACS system (Philips Inc). Where the prospective calcium score was not available, the aortic valve calcium score was calculated retrospectively. The reporting radiologist was blinded to clinical details including stroke status. The aortic valve calcium was calculated manually from sequential 3-mm axial slices, taking care to exclude extra valvular calcium, such as mitral annular, coronary calcification, and LVOT calcium, according to a methodology previously described.^9^ The presence or absence of LVOT calcium was separately recorded in a binary fashion. An example of an axial 3-mm aortic valve calcium slice from which the score was calculated is shown in Figure 1.

### TAVR procedure

The TAVR procedure was performed in a standard fashion via the transfemoral, transaxillary, transapical, or transcaval routes. Activated clotting time was kept above 250 seconds in all cases with intra-arterial heparin. No cerebral embolic protection devices (CEPDs) were used in this cohort.

### Statistics

Data were analyzed using an open-source statistical environment (R studio version 4.0.2). Normally distributed data are presented as mean and standard deviation. Non-normally distributed data are presented as median and interquartile range (IQR).

The relationship among aortic valve calcium score, sex, age, pre-existing comorbidities, procedure data, TAVR valve manufacturer, and stroke was assessed using univariate logistic regression models. In the event of multiple significant predictors, these would each be included in a multiple logistic regression model. We took this approach to minimize the number of covariates included in the model given we expected a relatively small number of events. For models with multilevel categorical predictors (device manufacturer), significance was reported using a Wald test across the levels.

## Results

All 497 patients who underwent a TAVR procedure at the Hammersmith Hospital between January 2017 and December 2019 were assessed for inclusion in the study. We excluded 32 patients who had CTs in other centers with no appropriate calcium score sequences for analysis. Twenty-three patients who had valve-in-valve procedures and 9 patients who had TAVR valve implantation in the mitral valve position were excluded. This left a final cohort of 433 patients.

### Baseline and procedural characteristics

The baseline characteristics of the cohort are shown in Table 1. The median age was 83 ​years (IQR, 78-87), and there were 228 (52.7%) male patients. One hundred and eight (24.9%) of the cohort were diabetic, 52 (12.0%) had a previous stroke or transient ischemic attack, and 127 (29.3%) had a history of atrial fibrillation (AF) or flutter. Fifty-six (12.9%) patients had a history of PAD, and 33 (7.6%) patients had a history of myocardial infarction.

**Table 1:** Baseline characteristics.

	*n* ​= ​433
Age, y	83 (78-87)
Male sex	228 (52.7%)
Diabetic	108 (24.9%)
Previous stroke or transient ischemic attack	52 (12.0%)
Creatinine, μmol/L	87 (72-110)
Previous myocardial infarction	33 (7.6%)
Peripheral arterial disease	56 (12.9%)
Atrial fibrillation or atrial flutter	127 (29.3%)
Edwards valve	244 (56.4%)
Medtronic valve	183 (42.3%)
Portico valve	6 (1.39%)
Procedure time, min	120 (95-160)
CT aortic valve calcium score, AU	2145 (1427-3247)

Data presented as *n* (%) or median (interquartile range).

AU, Agatston units; CT, computed tomography.

The TAVR procedure was performed in a standard fashion via the transfemoral (404, 93.3%), transaxillary (3, 0.7%), transsubclavian (19, 4.4%), transapical (5, 1.2%), and transcaval (2, 0.5%) routes. Three hundred and ninety (90.0%) were under light sedation, and 43 (9.9%) were under general anesthetic or converted to general anesthetic. Activated clotting time was kept above 250 seconds in all cases with intra-arterial heparin. One hundred fifty-four (35.6%) patients were anticoagulated prior to the TAVR.

### Stroke data

There were 22 patients who had a stroke prior to discharge from hospital (5.1%). Twenty-one (95.5%) strokes were infarctions; 1 (4.5%) was caused by an aortic dissection. There were no hemorrhagic strokes. Ten (45.5%) of the strokes were multiterritory and embolic in nature. Four (18.2%) were diagnosed in the cardiac catheter laboratory. The median time from end of the TAVR case until stroke diagnosis was 469 ​minutes (IQR, 127-1768).

Patients who had a stroke had a similar age to patients in the no-stroke group (median age of the stroke group was 83.5 ​years [IQR, 73.3-88.0], and the median age in the non-stroke stroke was 83.0 [IQR, 78-87]). There was a higher proportion of men in the stroke group than in the no-stroke group (14/22, 63.6% in the stroke group; 214/411, 52.1% in the no-stroke group). Among patients who had a stroke, 11 of 22 had an Edwards valve (50.0%), and 11 of 22 cases had a Medtronic valve (50.0%). The procedure time was significantly longer in stroke patients (median procedure time 168 ​minutes [IQR, 128-211]) than that in nonstroke patients (120 ​minutes [IQR, 94-154]).

### Aortic valve calcium score

**Figure 2:**
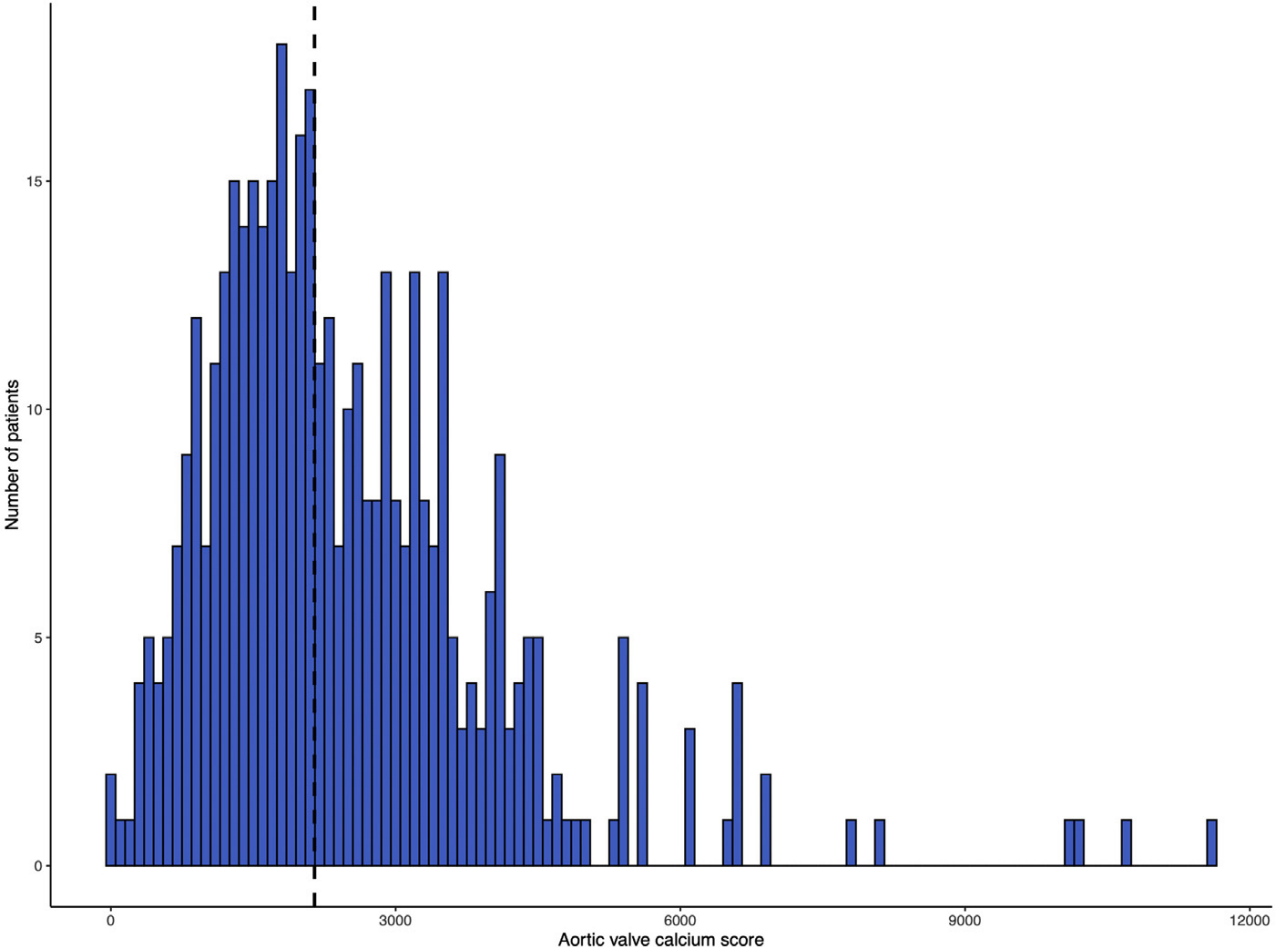
The distribution of aortic valve calcium score in the transcatheter aortic valve replacement cohort. The dashed line represents the study median.

The median aortic valve calcium score in the whole cohort was 2145 Agatston units (AU) (IQR, 1427-3247). The median aortic valve calcium score was significantly higher in patients who had stroke (2727 AU [IQR, 1526-3547]) than that in patients who did not have a stroke (2140 AU [IQR, 1426-3189]). The distribution of aortic valve calcium scores in the whole cohort can be seen in Figure 2.

### Logistic regression

**Figure 3:**
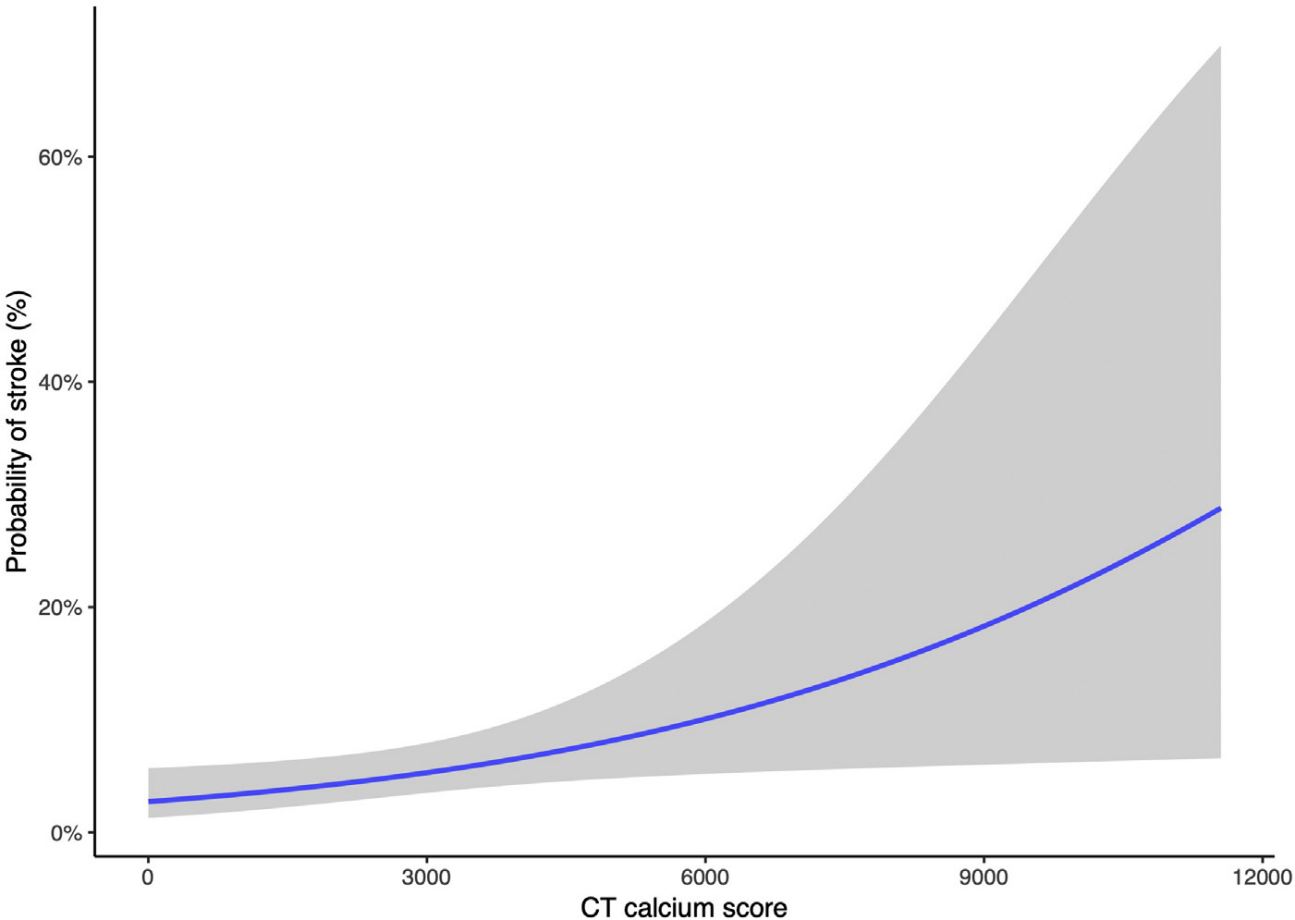
Logistic regression analysis showing the association of CT aortic valve calcium score and probability of stroke. The gray area represents the 95% confidence interval. CT, computed tomography.

In a logistic regression model (Table 2), the aortic valve calcium score was independently associated with stroke (odds ratio [OR], 1.26; 95% confidence interval [CI], 1.01-1.53; *P* = .02). Higher aortic valve calcium scores were associated with higher stroke risk. The logistic regression showing probability of stroke by aortic valve calcium score is shown in Figure 3. The presence or absence of LVOT calcification did not independently predict stroke (*P* ​= ​.68), and the addition of LVOT calcification to the aortic valve calcium model did not incrementally predict stroke (*P* = .52). PAD was significantly associated with stroke in a regression model (OR, 4.32; 95% CI, 1.65-10.65; *P* = .0018). When incorporating the independent predictors of aortic valve calcium and PAD into a combined model, they remained significantly associated with stroke (aortic valve calcium *P* = .032 and PAD *P* ​= ​.0023). A longer procedure time was also significantly associated with stroke (OR, 1.01; 95% CI, 1.00-1.02; *P* = .00057).

**Table 2:** Logistic regression.

Predictor	B, SE	95% CI for odds ratio for stroke			*P*
		Lower	Odds ratio	Upper	
CT aortic valve calcium score	0.2314 (0.103)	1.01	1.26	1.53	.025
Peripheral arterial disease	1.46 (0.4691)	1.65	4.32	10.65	.0018
Procedure time, min	0.01 (0.0031)	1.00	1.01	1.02	.00057
Age	−0.02911 (0.030)	0.92	0.97	1.03	.33
Male sex	0.4815 (0.4541)	0.68	1.61	4.11	.29
Diabetes	0.2099 (0.635)	0.41	1.62	5.35	.74

Results for the logistic regression models. Values are given for each predictor, with stroke as the outcome.

CT, computed tomography.

There was no difference in stroke across the 3 manufacturers in the study (*P* = .79). Age (*P* = .33), sex (*P* = .29), AF or flutter (*P* = .82), and diabetes (*P* = .74) were not significant predictors of stroke. The characteristics of stroke and no-stroke patients are given in Table 3.

**Table 3:** Stroke vs no-stroke patient characteristics.

	No stroke (*n* ​= ​411)	Stroke (*n* ​= ​22)
CT aortic valve calcium score	2140 (1426-3189)	2727 (1526-3547)
Age, y	83.0 (78-87)	83.5 (74.3-87.8)
Male sex	162 (39.4%)	14 (63.6%)
Diabetic	103 (25.1%)	4 (18.2%)
Previous stroke or TIA	48 (11.7%)	4 (18.2%)
Creatinine	87 (72-111)	93.5 (78-104)
Previous myocardial infarction	32 (7.8%)	1 (4.5%)
Extra-cardiac arterial disease	48 (11.7%)	8 (36.4%)
Atrial fibrillation or atrial flutter	121 (29.4%)	6 (27.3%)
Edwards valve	184 (44.8%)	11 (50.0%)
Medtronic valve	221 (53.8%)	11 (50.0%)
Procedure time, min	120 (94-154)	168 (128-211)

Data presented are *n* (%) or median (interquartile range).

CT, computed tomography; TIA, transient ischemic attack.

### Sex-stratified data

Male (52.7%) and female patients were of similar age, had similar rates of diabetes, previous stoke, and AF or flutter (Table 4). Male patients had higher rates of previous myocardial infarction (11.4% in male patients, 6.3% in female patients) and PAD (18.9% of male patients, 7.3% of female patients). The median aortic valve calcium score in male patients was significantly higher (2608 AU [IQR, 1733-3703 AU] vs 1826 [IQR, 1109-2681 AU]) than that in female patients (*P* ≤ .0001). Sex was not significantly associated with stroke (*P* ​= ​.29).

**Table 4:** Sex-stratified data

	Male (*n* ​= ​228)	Female (*n* ​= ​205)
Age, y	82 (76-87)	83 (79-87)
Diabetes	56 (24.6%)	52 (25.4%)
Previous stroke or TIA	27 (11.8%)	25 (12.2%)
Previous myocardial infarction	26 (11.4%)	13 (6.3%)
Extracardiac vascular disease	43 (18.9%)	15 (7.3%)
Atrial fibrillation or flutter	77 (33.8%)	63 (30.7%)
Edwards valve	119 (52.2%)	119 (58.0%)
Medtronic valve	106 (46.5%)	83 (40.5%)
Stroke	14 (6.1%)	8 (3.9%)
Median aortic valve calcium score, AU	2608 (1733-3703)	1826 (1109-2681)

Data presented as *n* (%) or median (interquartile range).

AU, Agatston units; TIA, transient ischemic attack.

## Discussion

In this retrospective, observational cohort study, we have demonstrated (1) a higher aortic valve calcium score, (2) PAD, and (3) longer procedural time are associated with stroke after TAVR.

### Aortic valve calcium score

TAVR has led to a paradigm shift in the treatment of patients with aortic stenosis, providing a treatment option for patients when none was previously available and also providing a lower risk alternative to surgery in certain cohorts of patients.^5^^,^^18^ The rapid recovery times and low complication rates mean that it is now challenging conventional surgery in even lower risk patients.^2^

Aortic valve calcium score is associated with more rapid aortic stenosis progression and is also used to help decisions with regard to intervention in patients with equivocal echocardiographic findings.^19^ Its role as a marker of periprocedural stroke, however, is less clear. Calcification in the LVOT has previously been associated with acute stroke in TAVR patients, but not the overall aortic valve calcium score.^20^

Of all the complications that can occur during cardiac procedures, stroke is the most feared by patients.^21^ The long-term impact of a stroke is unpredictable and may significantly reduce the patient’s quality of life. At present, there is no clear means of determining an individual patient’s stroke risk during TAVR. This study suggests that aortic valve calcium score is an independent risk factor for stroke; with higher aortic valve calcium scores predicting a higher risk (Central Illustration). Systematically identifying predictors of stroke will allow more refined discussions with patients with regard to their individual risk of stroke compared to population-based risks that are used to obtain consent from patients in clinical practice.

**Central Illustration:**
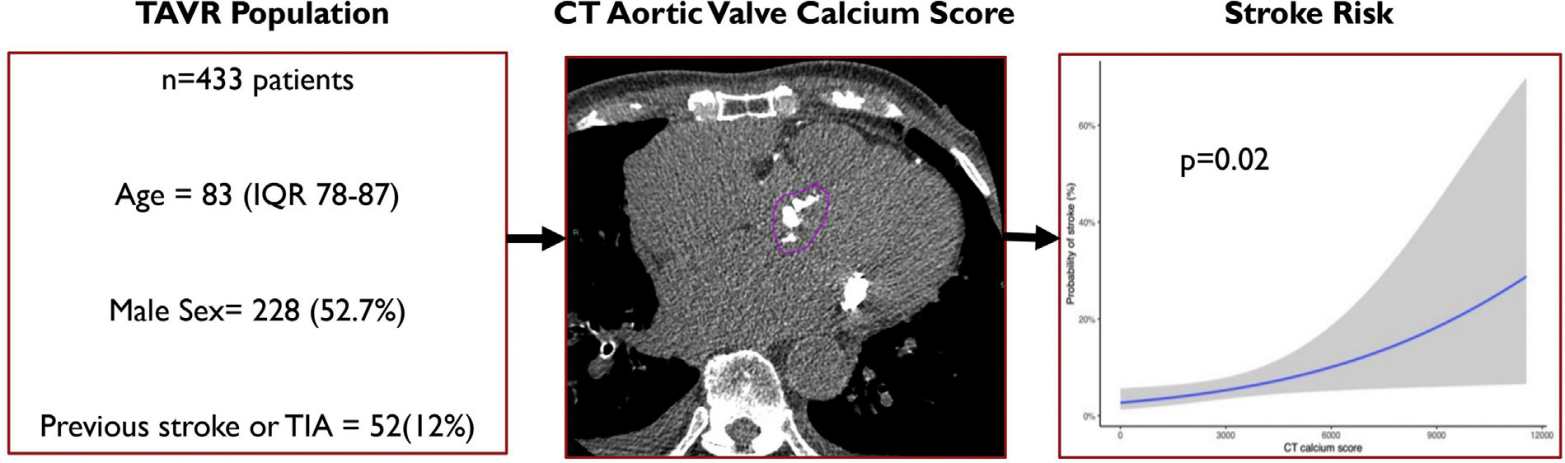
In this cohort of 433 patients, the aortic valve calcium score from the preprocedural cardiac CT was significantly associated with acute stroke after TAVR (*P* = .02). CT, computed tomography; TAVR, transcatheter aortic valve replacement.

### Aortic valve calcium score and stroke in lower risk populations

Randomized trial data have demonstrated that TAVR may be superior to SAVR in lower risk patients.^2^ This may lead to an increase in demand for TAVR in this patient cohort. These patients are at low risk due to younger age and lower comorbidity, often expecting to return to a more active lifestyle and live for many years after valve insertion. The relative impact of stroke on their quality of life is therefore arguably greater than that in older and more comorbid higher risk populations, who will have a lower predicted lifespan.

An understanding of the relative risk of stroke following TAVR vs SAVR is therefore paramount when discussing the relative merits of these competing therapies. These relative risks on a population basis may be very different from a patient’s individual risk. Recent nonrandomized data have demonstrated a higher rate of stroke with SAVR than with TAVR in patients with previous stroke (8.0% vs 3.7%), with no difference seen in patients without previous stroke (2.1% vs 1.7%).^22^ A young patient with a high aortic valve calcium score may be at much higher risk of stroke after TAVR than a frail higher risk patient with a low aortic valve calcium score. A stroke in the younger patient may result in many years of dependent living, which could be avoided if predicted and the procedure with the lowest risk of stroke selected. Using predictors of stroke such as aortic valve calcium score will therefore allow us to have a more nuanced understanding of the risk of complication for each patient and help arrive at the most appropriate intervention for the individual patient in clinic. In light of the association of aortic valve calcium score with stroke in a TAVR population, a similar association should be investigated in patients undergoing SAVR to contribute to this discussion.

### Aortic valve calcium may help refine the use of embolic protection devices

Identifying that a patient is at higher risk of stroke may also allow physicians to adapt the TAVR procedure to minimize risk. CEPDs have been used as an adjunct to TAVR in an attempt to reduce the incidence of acute stroke. A recent large registry study found a signal suggesting small reduction in stroke risk with CEPD in a propensity-matched analysis.^23^ However, a meta-analysis of 16 trials of CEPDs including all comers found no overall reduction in clinically evident stroke or mortality at 30 ​days.^24^ The benefit of CEPDs in higher stroke risk patients may have been masked by the inclusion of patients at low risk of stroke. It is therefore plausible that aortic valve calcium score can be used to target the patients in which CEPDs may have the most benefit. A large prospective randomized trial of CEPDs in TAVR patients with a high aortic valve calcium score would address this question.

### PAD and procedure times

In our study, acute stroke after TAVR was associated with patients with PAD and longer procedure times. Both PAD and procedural time have been found to be predictors of stroke in previous studies.^25^, ^26^, ^27^ Longer procedure time may reflect a complication during the procedure such as valve embolization, the need to balloon valvuloplasty before or after valve insertion, or a more difficult procedure in anatomically complex patients with no causal relationship.^6^ Given that 18.2% of the strokes in this cohort were diagnosed during the procedure, the longer procedure time may also be *a consequence* of stroke, rather than being predictive of it.

### Study limitations

This single-center, retrospective cohort study has demonstrated an association between aortic valve calcium score and stroke. While we have attempted in this study to correct for confounders, such as age and comorbidity, this association may not be causal. The findings of this study would be strengthened by replication in a prospective trial including multiple centers, and this will be the focus of future work. Additionally, as a retrospective study, the criteria used for post-TAVR neuroimaging is not standardized which may have influenced the diagnosis of stroke in the cohort.

This study was designed to detect periprocedural and early stroke, and as such, we curtailed follow-up at the point of discharge. As such, the effect of aortic valve calcium score on later strokes cannot be commented upon.

The majority of aortic valve calcium scores in this cohort were reported in a single center, which could have impacted the reproducibility of these data.^28^ However, aortic valve calcium score is highly reproducible (the Philips system for Agatston scoring of the aortic valve has an intraobserver variability of 9 ​± ​42 AU).^29^ These findings would be strengthened by replication in other centers and multicenter cohorts.

## Conclusion

Aortic valve calcium score from pre-TAVR CT is an independent risk factor for stroke in the TAVR population. This is an additional clinical value of the pre-TAVR aortic valve calcium score and should be considered when discussing periprocedural stroke risk.

## Declaration of competing interest

Dr Foley and Dr Al-Lamee report a relationship with Menarini International Pharmaceutics that includes speaking and lecture fees. Dr Al-Lamee, Dr Sen, Dr Petraco, and Dr Foley report a relationship with Philips Healthcare that includes speaking and lecture fees. Dr Rana reports a relationship with Occlutech GmbH and Holistick Medical that includes consulting or advisory and speaking and lecture fees. All other authors report no conflicts to disclose.
